# TNF-α Preconditioning Improves the Therapeutic Efficacy of Mesenchymal Stem Cells in an Experimental Model of Atherosclerosis

**DOI:** 10.3390/cells12182262

**Published:** 2023-09-13

**Authors:** Aliya Sekenova, Yelena Li, Assel Issabekova, Arman Saparov, Vyacheslav Ogay

**Affiliations:** 1 Laboratory of Stem Cells, National Center for Biotechnology, Astana 010000, Kazakhstan; 2 Department of Medicine, School of Medicine, Nazarbayev University, Astana 010000, Kazakhstan

**Keywords:** mesenchymal stem cells, preconditioning, TNF-α, atherosclerosis

## Abstract

Atherosclerosis (AS) is an inflammatory disease involving multiple factors in its initiation and development. In recent years, the potential application of mesenchymal stem cells (MSCs) for treating AS has been investigated. This study examined the effect of TNF-α preconditioning on MSCs’ therapeutic efficacy in treating AS in ApoE KO mice. TNF-α-treated MSCs were administered to high-fat diet-treated ApoE KO mice. Cytokine and serum lipid levels were measured before and after treatment. Cryosections of the atherosclerotic aorta were stained with Oil-Red-O, and the relative areas of atherosclerotic lesions were measured. The level of Tregs were increased in TNF-α-MSC-treated animals compared to the MSCs group. In addition, the systemic administration of TNF-α-MSCs to ApoE KO mice reduced the level of proinflammatory cytokines such as TNF-α and IFN-γ and increased the level of the immunosuppressive IL-10 in the blood serum. Total cholesterol and LDL levels were decreased, and HDL levels were increased in the TNF-α-MSCs group of ApoE KO mice. A histological analysis showed that TNF-α-MSCs decreased the size of the atherosclerotic lesion in the aorta of ApoE KO mice by 38%, although there was no significant difference when compared with untreated MSCs. Thus, our data demonstrate that TNF-α-MSCs are more effective at treating AS than untreated MSCs.

## 1. Introduction

Atherosclerosis (AS) is the most widespread vascular disease, caused by the formation of plaques consisting of cholesterol and lipids inside vessels, which lead to arterial blockage and blood supply reduction to organs and tissues [1]. In this regard, AS causes cardiovascular diseases, leading to myocardial infarction, stroke, ischemia of the lower extremities, aneurysms, angina pectoris, cerebral ischemic attacks, ischemic insults, endothelial dysfunction, the disruption of peripheral arteries, plaque rupture, hypertension, and thrombosis [2,3,4]. Despite available therapy designed to lower the level of lipids and cholesterol in patients’ blood, AS is the most prevalent reason for physical impairment and untimely death worldwide [4,5].

The pathogenesis of AS is primarily associated with inflammatory processes involving cells of the adaptive and innate immune systems [4,5,6,7]. The dysfunction of endothelial cells is caused by the accumulation of ox-LDL (oxidized low-density lipoprotein), which is phagocytosed by macrophages and leads to their subsequent transformation into foam cells. Activated macrophages and foam cells produce several proinflammatory cytokines that stimulate endothelial cells to upregulate integrins and selectins on their surface, which mediates the recruitment of leukocytes and T and B cells to the walls of the vessels [8].

Currently, pharmaceuticals such as statins are a therapeutic approach to treating AS [9]. Nonetheless, regardless of the advances established in pharmaceutical therapy using statins, a few extensive case–control clinical studies have demonstrated that almost two-thirds of patients treated with statins suffer from cardiovascular complications [10]. Furthermore, most patients cannot endure continuing treatment with statins to attain favorable low-density lipoprotein (LDL) levels [9]. Hence, to improve the efficacy of AS therapy, additional studies should be performed.

Cell-based therapy using MSCs is a new approach to treating AS [11,12,13,14]. MSCs are a fibroblast-like population of cells that can be isolated from various human organs and tissues, including but not limited to the adipose tissue, bone marrow, skeletal muscle, synovium, umbilical cord, and others [15,16]. Scientists and clinicians have been interested in MSCs due to their potential for multilinear differentiation, moderate immunogenicity, and ability to regenerate various tissues and organs [17]. In addition, recently published studies have demonstrated that MSCs possess robust immunosuppressive and immunomodulatory features, which are mediated by both cell–cell contact and the production of cytokines, growth factors, and mediators, such as TGF-β (transforming growth factor β), HGF (hepatocyte growth factor), PGE2 (prostaglandin E2), a soluble form of HLA-G5 (human leukocyte antigen G5), IDO (indoleamine 2,3-dioxygenase), iNOS (inducible nitric oxide synthase), and others [13,18,19]. MSCs can suppress the function of proinflammatory M1 macrophages, dendritic cells, T and B cells, and natural killer cells and promote the formation of immune cells with anti-inflammatory abilities [19,20].

Due to MSCs’ immunomodulatory characteristic, these cells’ beneficial potential in treating AS has been extensively investigated in experimental models such as apolipoprotein E (*Apoe gene*) knockout (Apoe^−/−^ or ApoE KO) and low-density lipoprotein receptor (*Ldlr gene*) knockout (Ldlr^−/−^ or LDLr KO) mice [11,12,13,21]. Several studies have reported that MSCs can exert immunomodulatory, regenerative, and athero-protective effects in experimental AS models [13]. For instance, MSCs can reportedly transfer to areas of the endothelium with atherosclerotic lesions and differentiate into endothelial cells, as well as suppress the dysfunction of endothelial cells and plaque formation [22]. Moreover, MSCs not only enhance the endothelial recovery and composition of collagen fibers in blood vessel walls but can also stabilize vulnerable plaques in AS by suppressing inflammatory processes and cell apoptosis in atherosclerotic plaques [23,24]. The transplantation of allogeneic MSCs is known to significantly increase the production of anti-inflammatory cytokines, namely TGF-β1 and interleukin-10 (IL-10), and decrease the production of proinflammatory cytokines and chemokines such as TNF-α (tumor necrosis factor), interleukin-6 (IL-6), interleukin-1β (IL-1β), and monocyte chemoattractant protein 1 (MCP-1) [23,24]. Moreover, MSCs promote the development and generation of anti-inflammatory CD4^+^ CD25^+^ Foxp3^+^ (Forkhead box P3) regulatory T cells (Tregs), which decrease the level of macrophages and effector T cells along with the formation of foam cells in blood vessels [25]. In addition to inhibiting inflammatory processes, MSCs can notably diminish LDL and cholesterol levels in the blood serum of experimental animals [21]. Thus, MSCs’ application has multifactorial therapeutic potential that can reduce inflammatory responses, dyslipidemia, and atherosclerotic plaque generation.

New approaches and strategies to improve the immunomodulatory and regenerative capacities of MSCs in treating AS should be further investigated. One such strategy is preconditioning under ex vivo conditions in a defined environment using various biological, chemical, or physical factors [13,26,27,28]. To improve the therapeutic efficacy of MSCs, variable preconditioning approaches have been suggested, including but not limited to cytokines, hypoxia, a 3D culture, the genetic modification of MSCs, and autophagy modeling [13,26,27]. Hypoxic stimulation and cytokines are two main strategies used to enhance the immunomodulatory features of MSCs [13,27,29]. Therefore, the preconditioning of MSCs using proinflammatory cytokines such as IFN-γ, TNF-α, IL-1β, and interleukin-17A (IL-17A) also beneficially enhanced the immunomodulatory properties of MSCs, as demonstrated by *in vitro* and *in vivo* studies [30,31,32,33].

Preconditioning with TNF-α modulates proinflammatory or Th-1-like immune environments that activate MSCs to produce factors and bioactive trophic molecules [20]. For example, the high secretion of IDO promoted the generation of anti-inflammatory M2 macrophages and decreased the proliferation of peripheral blood mononuclear cells [33]. TNF-α can regulate the expression of chemokines (interleukin 8 (IL-8), C-C motif ligand 2 (CCL2), C-C motif ligand 3 (CCL3), C-C motif ligand 5 (CCL5)), interleukins (interleukin 1 (IL-1), IL-6, and IL-7), immunosuppressive molecules (PGE2 and IDO), and factors responsible for angiogenesis (vascular endothelial growth factor (VEGF)) [31,34]. Moreover, conditions with high levels of TNF-α and IFN-γ reportedly activate MSCs to secrete high levels of immunomodulatory mediators promoting Tregs generation [20]. However, in conditions with low levels of TNF-α and IFN-γ, MSCs can secrete low levels of immunomodulatory mediators promoting the activation of immune cells and the recruitment of lymphocytes [20]. Preconditioning adipose-derived MSCs with TNF-α (10 ng/mL) improves wound closure by promoting angiogenesis and the migration of immune cells [34]. The therapeutic effects of TNF-α-preconditioned MSCs (TNF-α-MSCs) are mediated by a cell–cell contact mechanism, exosomal miR1260b in the Wnt family member 5A-connected (Wnt5a) receptor activator of nuclear factor kappa-B ligand (RANKL) pathway, or paracrine activity on the nuclear factor kappa-light-chain-enhancer of activated B cells (NF-kB) pathway or nitric oxide (NO) production [12,34,35,36].

Thus, based on previously published data, we further studied the therapeutic potential of TNF-α-preconditioned MSCs on atherosclerotic plaques in our experimental model of AS using ApoE KO mice.

## 2. Materials and Methods

### 2.1. Animals

Male C57BL/6 mice aged 2–3 weeks’ old were purchased from the SPF-vivarium of Masgut Aikimbayev’s National Scientific Center for Especially Dangerous Infections at the Ministry of Health of the Republic of Kazakhstan (Almaty, Kazakhstan). Male ApoE KO mice (B6.129P2-Apoe^tm1Unc/J^) approximately 2–3 weeks old were also obtained from the Jackson Laboratory (Bar Harbor, ME, USA). The animals were kept in a temperature-controlled environment (23 °C) with 60% relative humidity, a 12 h light/dark cycle, and *ad libitum* access to food and water. Animal experiments were conducted following the European Convention for the Protection of Vertebrate Animals (ETS 123) and approved by the Local Ethical and Institutional Review Board of the National Center for Biotechnology (order №6, 15 November 2017).

### 2.2. MSC Isolation and Characterization

We isolated MSCs from mouse compact bones according to Zhu et al. [37]. The tibias and femurs were dissected from the hind limbs of mice and cleaned out of muscles. Once the ends of the femurs and tibias were cut, the bone marrow was flushed out with α-MEM (α—minimum essential medium) (Gibco, Waltham, MA, USA). Femurs and tibias were cut into small pieces and placed in α-MEM containing 10% fetal bovine serum (FBS) (Gibco, Waltham, MA, USA) and 0.1% collagenase II (Gibco, Waltham, MA, USA) for 2 h in a shaking incubator at 37 °C for 200 rpm. Then, small pieces of the femur and tibia were washed with α-MEM and seeded into a T25 culture flask in α-MEM with 10% FBS, 1% antibiotic–antimycotic (Gibco, Waltham, MA, USA), and a 1% Non-Essential Amino Acids Solution (Gibco, Waltham, MA, USA), (MSCs’ medium). After incubation for 3–5 days, small pieces of femurs and tibias and non-adherent cells were removed. Then, the cells were cultured and passaged for further experiments.

Colony-forming unit-fibroblast assay CB MSCs (passage 3) were harvested using TrypLE Express (Gibco, Waltham, MA, USA) and seeded into T25 culture flasks (Corning, New York, NY, USA) at a 1000 cells/flask density. After 14 days, cell cultures were washed with phosphate-buffered saline (PBS) (Thermo Fisher Scientific, Waltham, MA, USA) and stained with 0.5% crystal violet (Sigma, St. Louis, MO, USA) in methanol (Sigma, St. Louis, MO, USA) for 10 min at room temperature. After staining, the flasks were washed with PBS, allowed to dry, and colonies were counted and analyzed using a stereomicroscope SZ61 (Olympus, Hamburg, Germany).

We used a multilineage differentiation assay. MSCs in a quantity of 1 × 10^4^ cells/cm^2^ were seeded in a 6-well culture plate (Corning, New York, NY, USA). An adipogenic differentiation assay was performed according to the protocol in [38]. After 21 days, cells were fixed with a 4% paraformaldehyde solution (PFA) (Sigma, St. Louis, MO, USA) and stained with Oil-Red-O (Sigma, St. Louis, MO, USA). An osteogenic differentiation assay was conducted according to the protocol in [38]. After 21 days, differentiated cells were evaluated using Alizarin (Sigma, St. Louis, MO, USA) red staining. A chondrogenic differentiation assay was performed according to the protocol [38]. To create chondrogenic micromass pellets, 2.5 × 10^5^ cells were placed in a 96-well polypropylene plate (Corning, New York, NY, USA), centrifuged at 500× *g*, and placed in an incubator at 37 °C and 5% CO_2_. The medium was changed twice a week. After 3 weeks, the cell pellets were harvested, fixed with 10% neutral-buffered formalin (Sigma, St. Louis, MO, USA), paraffin-embedded, sectioned at 5 µm, and stained with Toluidine blue (Sigma, St. Louis, MO, USA).

We used flow cytometry to phenotype the obtained MSCs with the following antibodies: CD29-PE (clone HM β1-1), CD31-PE (clone MEC 13.3), CD44-FITC (clone IM7), CD45-FITC (clone 30-F11), CD90-FITC (clone 53-2.1), and CD105-PE (clone MJ7/18) (all purchased from BD Biosciences, Franklin Lakes, NJ, USA). A flow cytometry analysis was performed on an Attune NxT Acoustic Focusing Flow Cytometer (Thermo Fisher Scientific, Waltham, MA, USA). Obtained data were analyzed using FlowJo_v10.7.2 software (FlowJo LLC, Franklin Lakes, NJ, USA).

### 2.3. Cytokine Preconditioning of MSCs

For preconditioning, 2 × 10^5^ cells were plated in T25 culture flasks and cultured in an MSC medium for 2 days at 37 °C and 5% CO_2_. Then, MSCs were cultured in the presence of 20 ng/mL of TNF-α (PMC3014; Thermo Fisher Scientific, Waltham, MA, USA) for 24 h at 37 °C and 5% CO_2_ (carbon oxide). The cytokine concentration was selected according to previously published data [25,35,36,37,39,40,41].

### 2.4. Atherosclerosis

**Figure 1:**
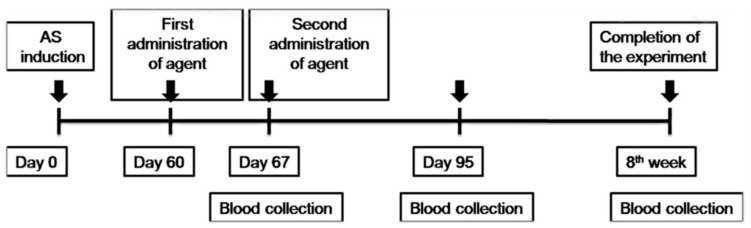
Schematic illustration of the study design. TNF-α-MSCs and MSCs (untreated) were administered with two *i.v*. (within 1 week of the interval) injections in a mouse model of AS. MSCs, mesenchymal stem cells; TNF-α-MSCs, TNF-α-preconditioned MSCs; AS, atherosclerosis; *i.v.*, intravenous.

To induce atherosclerosis (Figure 1), ApoE KO mice were fed daily with an atherogenic diet containing 0.15% cholesterol and 0.21% butter oil (Western diet E-15721-34; SSNIFF Spezialdiäten GmbH, Soest, Germany). After 8 weeks, the aorta was isolated and stained with Oil-Red-O (Sigma, St. Louis, MO, USA) to determine the presence of atherosclerotic plaques. ApoE KO mice (five mice per group) were administered two *i.v.* (intravenous) injections of 1 × 10^6^ TNF-α-MSCs, 1 × 10^6^ MSCs (untreated), and the same amount of liquid PBS (phosphate-buffered saline) within 1 week of the interval. Figure 1 presents the design scheme of the protocol.

### 2.5. Flow Cytometry

At the end of the experiment, spleens were isolated from ApoE KO mice and placed in a chilled RPMI-1640 medium (Gibco, Carlsbad, CA, USA), mashed through a 70 µm nylon cell filter (Corning, Corning, NY, USA), and centrifuged at 400× *g* for 5 min. The obtained splenocytes were stained with relevant antibodies. The following antibodies were used: CD4-PE (553048, PE Rat Anti-Mouse CD4, BD Biosciences, Franklin Lakes, NJ, USA), CD8-PE (553032, PE Rat Anti-Mouse CD8a, BD Biosciences, USA), and FoxP3-Alexa Fluor 488 (560403, Alexa Fluor 488 Rat anti-Mouse Foxp3, BD Biosciences, USA). The following isotype match antibodies were used: IgG2a-PE (551799, PE Rat IgG2a, k Isotype Control, BD Biosciences, USA), and IgG2b-FITC (556923, FITC Rat IgG2b, k Isotype Control, BD Biosciences, USA). A flow cytometry analysis was performed on a Beckman Coulter Flow Cytometer (BD Biosciences, USA). Obtained data were analyzed using FlowJo software (FlowJo LLC, Franklin Lakes, NJ, USA).

### 2.6. Cytokines

Serum levels of IL-6 (555240, Mouse IL-6 ELISA Set, BD Biosciences, Franklin Lakes, NJ, USA), IL-10 (555252, Mouse IL-10 ELISA Set, BD Biosciences, USA), TNF-α (555268, Mouse TNF-α ELISA Set, BD Biosciences, USA), and IFN-γ (555138, Mouse IFN-γ ELISA Set, BD Biosciences, USA) were detected in five animals per group with enzyme-linked immunosorbent assay (ELISA) kits according to the manufacturer’s instructions.

### 2.7. Serum Cholesterol Levels

During the experiment, blood samples were collected from tail vein bleeding. Serum levels of cholesterol, LDL, and high-density lipoprotein (HDL) were determined using an enzymatic analysis (ab65390, Cholesterol Assay Kit; Abcam, Cambridge, UK), according to the manufacturer’s instructions.

### 2.8. Spleens’ Weight Evaluation

After 8 weeks of the treatment period, mice were deduced from the experiment in a CO_2_ box. After dissection, the spleens were weighed by scale (OHAUS Pioneer PA214C, Shanghai, China) and photographed. Calculations of the spleens’ weights (in %) were conducted for every group of experimental mice.

### 2.9. Histological Analysis

Samples were stored in 10% formalin and transferred to 20% sucrose (Sigma, St. Louis, MO, USA) for 24 h. Aortic samples were embedded in OCT (optimal cutting temperature compound) (Sakura Finetek, Torrance, CA, USA) for 10 min, and incubated at −70 °C for 24 h. The 15 µm cryosections were prepared from embedded aorta specimens on a microtome (MNT SLEE, NIeder-Olm, Germany). To determine the presence of atherosclerotic plaques, cryosections of the aorta were stained with Oil-Red-O (Sigma, St. Louis, MO, USA). Using the ImageJ 1.53k program (U.S. NIH, Bethesda, ML, USA), the total area of the aortic vessel and the area without lesions were determined. Afterward, the mean lesion size of the atherosclerotic lesion was calculated by deducting the area without lesions from the total area of the vessel, dividing the result by the total area, and multiplying it by 100% [42]. All images were analyzed in Zen program of Axio Observer inverted microscope A1 (Carl Zeiss, Oberkochen, Germany) and by ImageJ 1.53k software.

### 2.10. Statistical Analysis

Data are presented as the mean ± standard deviation (SD). Values of three or more samples were compared with a one-way ANOVA and two groups with values for more than one variable were determined with a two-way ANOVA. The statistical analysis was performed using GraphPad Prism 9.1.0 software (GraphPad Software, San Diego, CA, USA). Probability values *p* < 0.05 were considered significant.

## 3. Results

### 3.1. Culture and Characterization of MSCs

MSCs were obtained from compact bone and showed typical MSC characteristics, which were confirmed with the flow cytometry analysis. Obtained MSCs showed the capacity to form fibroblast-like colonies and the potential to differentiate into adipocytes, osteoblasts, and chondrocytes. They also expressed MSC markers CD29, CD44, CD90, and CD105 (Supplementary Figure S1).

### 3.2. Determination of AS Plaque

The presence of atherosclerotic plaques in the isolated aorta was determined with Oil-Red-O staining (Supplementary Figure S2). We observed lipid deposition on the inner side of the aorta’s vessel wall in ApoE KO mice fed an atherogenic diet.

### 3.3. Analysis of T-Cell Subpopulations with Flow Cytometry

**Figure 2:**
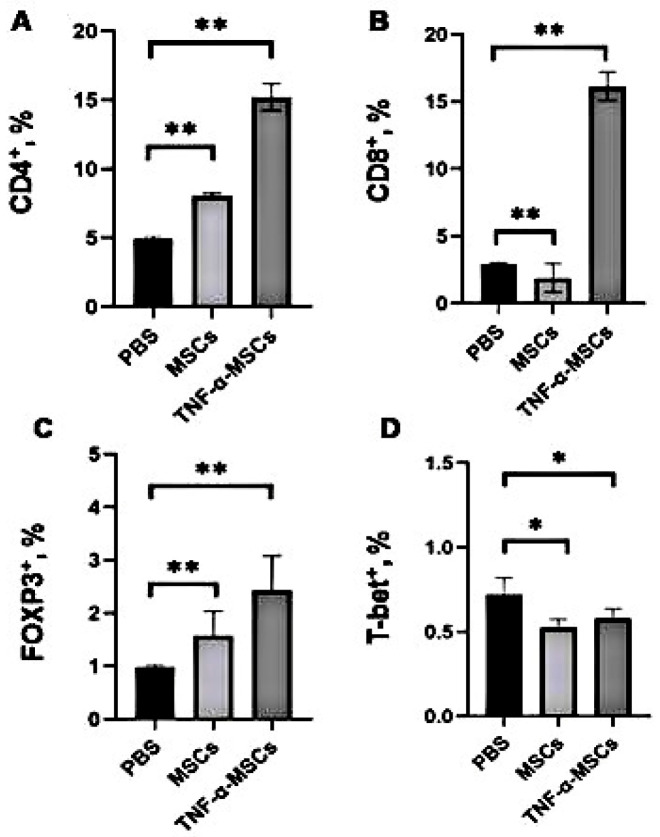
TNF-α-MSCs treatment affects CD4⁺T cells’, CD8⁺T cells’, and FOXP3⁺Tregs’ responses in vivo. Male ApoE KO mice obtained two *i.v*. injections of either PBS (control), 1 × 10^6^ MSCs (MCSs), or 1 × 10^6^ TNF-α-preconditioned MSCs (TNF-α-MSCs) within 1 week of the interval. After 8 weeks, (**A**) CD4⁺T cells, (**B**) CD8⁺T cells, (**C**) FOXP3⁺Tregs, and (**D**) T-bet^+^ T cells in the spleen were determined with flow cytometry. All values are expressed as the mean ± SD and represent five mice per group. * *p* < 0.05, ** *p* < 0.01.

To determine whether MSCs can regulate the immune response and development of atherosclerosis, ApoE KO mice were administered two intravenous injections of MSCs and TNF-α-MSCs in a 1-week period. After 8 weeks of treatment, the number of CD4⁺ T cells in the TNF-α-MSCs group significantly increased compared to both MSCs and control groups (control: 5% versus TNF-α-MSCs: 15%; *p* < 0.01). Treatment with MSCs alone also significantly increased the number of CD4⁺ T cells (control: 5% versus MSCs: 8%; *p* < 0.01; Figure 2A). Similar changes were detected in the number of CD8⁺ T cells after injections of TNF-α-MSCs compared to the vehicle group. Furthermore, the use of MSCs alone decreased the number of CD8⁺ T cells (control: 3% versus MSCs: 2%; *p* < 0.01; Figure 2B). Moreover, more than a two-fold rise was detected in the number of spleen FOXP3⁺Tregs in the TNF-α-MSCs group compared to the control (control: 1% versus TNF-α-MSCs: 2.5%; *p* < 0.01; Figure 2C). Treatment with MSCs alone caused a similar increase in the number of FOXP3⁺Tregs but of a lesser magnitude. In contrast, treatment with both TNF-α-MSCs and MSCs alone significantly decreased the number of spleen T-bet⁺ Th1 cells (Figure 2D).

### 3.4. Analysis of Cytokine Profile of T Cells

**Figure 3:**
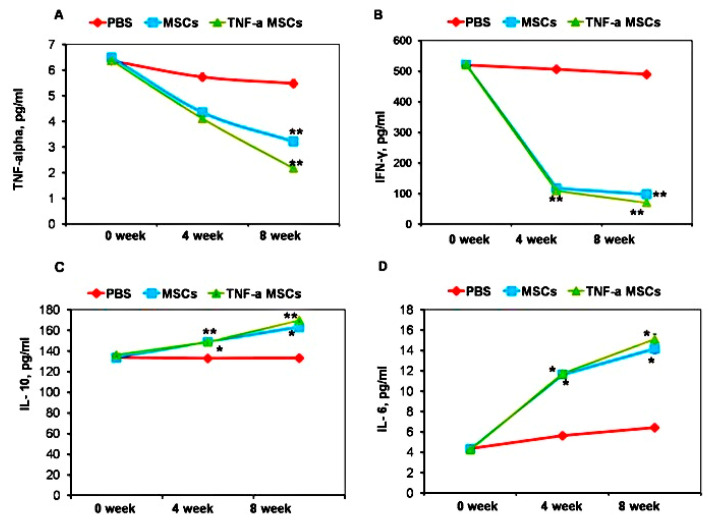
TNF-α-MSCs treatment reduces proinflammatory cytokine responses in vivo. (**A**) Serum TNF-α, (**B**) IFN-γ, (**C**) IL-10, and (**D**) IL-6 were measured with ELISA. Serum cytokine levels represent n = 5 pooled samples (0 days), n = 4 control, n = 5 MSC-treated, and n = 5 TNF-α-MSC-treated (4 weeks and 8 weeks) of all mice. * *p* < 0.05; ** *p* < 0.01.

To investigate whether MSCs exert immunomodulatory effects, we measured the cytokine profile before treatment and during the fourth week and eighth week of treatment. Treatment with both TNF-α-MSCs and MSCs significantly decreased the levels of circulating TNF-α at 8 weeks (*p* < 0.01, Figure 3A). Injections of both TNF-α-treated and untreated MSCs showed an even more profound effect on IFN-γ levels (Figure 3A). The levels of this cytokine were significantly decreased at both time points, namely 4 and 8 weeks after treatment. By contrast, treating atherosclerotic mice with both TNF-α-MSCs and MSCs alone significantly increased serum IL-10 (Figure 3C) and IL-6 (Figure 3D) levels at both time points, namely 4 and 8 weeks after treatment.

### 3.5. Lipid Analysis

**Figure 4:**
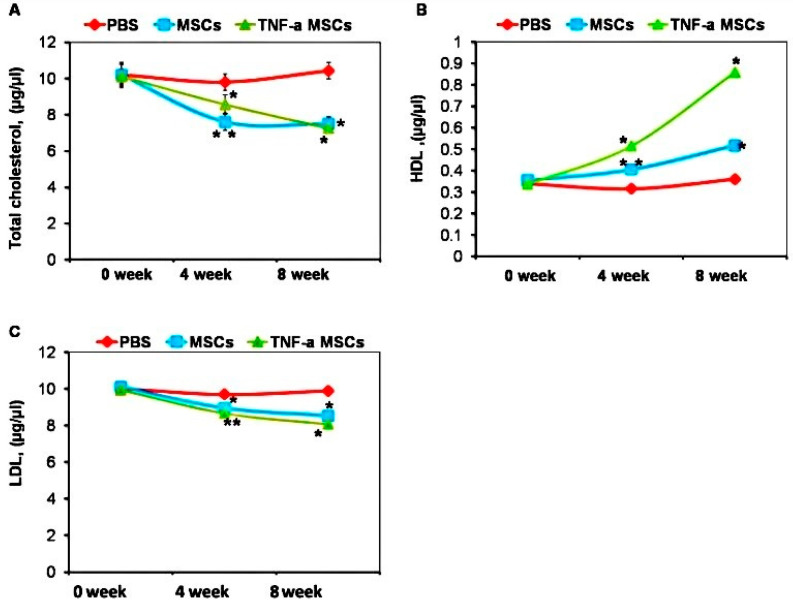
TNF-α-MSCs treatment increases HDL production in vivo. (**A**) Cholesterol, (**B**) HDL, and (**C**) LDL levels were observed during the experiment and measured with ELISA. Serum lipid levels represent n = 5 pooled samples (0 days), n = 4 control, n = 5 MSC-treated, and n = 5 TNF-α-MSC-treated (4 weeks and 8 weeks) of all mice. Data are illustrated as the mean ± SD. * *p* < 0.05; ** *p* < 0.01.

Since dyslipidemia can cause atherosclerosis, we measured cholesterol levels in the serum of treated and untreated animals. Treatment with both TNF-α-MSCs and untreated MSCs significantly decreased total cholesterol levels at both time points (Figure 4A). The treatment also decreased LDL levels (Figure 4C). In contrast, HDL levels were increased at both time points after treatment (Figure 4B).

### 3.6. Spleens’ Weights of ApoE KO Mice

**Figure 5:**
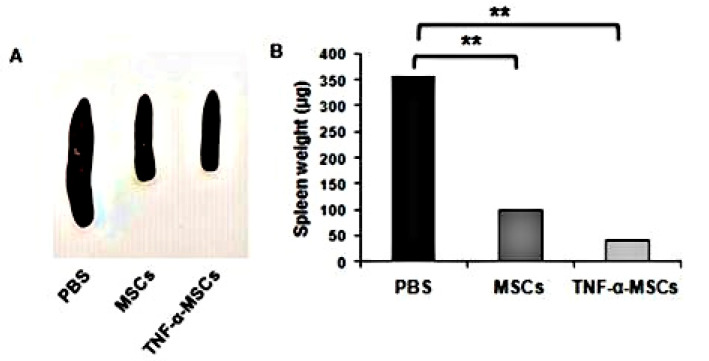
TNF-α-MSCs treatment reduces spleen weight in vivo. (**A**) After 8 weeks, we photographed representative images of spleens from PBS-, MSC-, and TNF-α-MSCs-treated ApoE KO mice. (**B**) Spleen weights represent n = 4 control, n = 5 MSC-treated, and n = 5 TNF-α-MSC-treated (8 weeks). Data are presented as the mean ± SD. ** *p* < 0.01.

Spleens from ApoE KO mice in the TNF-α-MSCs and MSCs groups significantly decreased in size compared to spleens from ApoE KO mice in the PBS group, which were enlarged until the eighth week of the experiment (Figure 5A). The spleen weights of the TNF-α-MSCs and MSCs groups were noticeably decreased compared to spleens from ApoE KO mice in the PBS group (Figure 5B).

### 3.7. Characterization of Aortic Cryosections Stained with Oil-Red-O Stain

**Figure 6:**
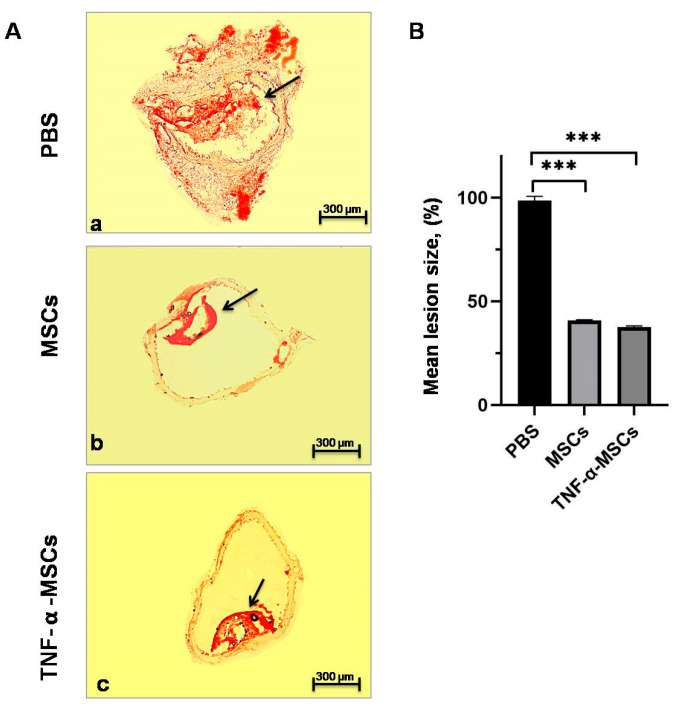
TNF-α-MSCs treatment reduces atherosclerotic lesion development. (**A**) Representative cross-sections of the thoracic aortic root stained with Oil-Red-O are demonstrated. (a) Representative image of thoracic aorta with atherosclerotic lesion of the control (PBS) group of ApoE KO mice stained with Oil-Red-O (red). (b) Representative image of thoracic aorta with atherosclerotic lesion of the MSCs group of ApoE KO mice stained with Oil-Red-O (red). (c) Representative image of thoracic aorta with atherosclerotic lesion of the TNF-α-MSCs group of ApoE KO mice stained with Oil-Red-O (red). Black arrow indicated the plaques with lipid content. Scale bar = 300 µm. (**B**) Mean lesion size was determined as a percentage of the total lesion area. Values are presented as the mean ± SD and represent all mice. *** *p* < 0.001.

TNF-α-MSCs treatment caused a considerable (*p* < 0.001, Figure 6B) reduction (38%) in atherosclerotic lesion size compared to control mice. Similar changes were found in the atherosclerotic lesion size of MSC-treated mice at 41% (*p* < 0.001, Figure 6B). The number of plaques with AS in the aorta section of MSCs and TNF-α-MSCs groups was significantly less (Supplementary Figure S3B,C) than in the aorta section of the PBS group. Notably, the size of the plaques with AS in the aorta section of MSCs and TNF-α-MSCs groups (Supplementary Figure S3B,C) were markedly small compared to plaques with AS in the aorta section of the PBS group. Plaque with lipid deposits (stained with Oil-red-O) was small in size in the aorta cross-section of the TNF-α-MSCs group (Supplementary Figure S4C, black arrow) compared to pronounced plaque with lipid deposits of the PBS group (Supplementary Figure S4A, black arrow). Also, some of the atherosclerotic plaques of the aorta cross-section of MSCs (Supplementary Figure S4B, blue arrow) and TNF-α-MSCs (Supplementary Figure S4C, blue arrow) groups did not contain the lipid deposits (stained with Oil-red-O) compared to aorta cross-section of the PBS group (Supplementary Figure S4A, black arrow). 

## 4. Discussion

One of the methods by which MSCs can mediate their therapeutic effects on immune-related diseases is immunomodulation [43,44]. Recent studies have reported that endothelial inflammation underlies vascular diseases such as atherosclerosis [45]. MSCs’ production of paracrine factors can improve endothelial dysfunction, inhibit inflammation, reduce dyslipidemia, inhibit vascular calcification, enhance plaque stability, and reduce the formation of foam cells and plaques [12,13]. On the other hand, preconditioning of MSCs with proinflammatory TNF-α creates an environment that mimics a Th1-similar (T helper 1) immune environment, which promotes MSCs’ secretion of bioactive trophic factors from their exosomes and microvesicles [20,43]. High concentrations of proinflammatory TNF-α and IFN-γ in MSCs activate high secretion levels of bioactive trophic factors that contribute to Treg generation and other immunomodulatory effects [43]. However, detailed mechanisms of how preconditioned MSCs can affect AS development have not been determined yet. Thus, this study explores the immunomodulatory effects of TNF-α-preconditioned MSCs on AS development.

The dosage of TNF-α (20 ng/mL) for MSCs’ preconditioning was based on previously published studies [27,39,40,41,43]. Previous studies reported that conditions with a low concentration of proinflammatory TNF-α and IFN-γ activate MSCs to secrete low levels of paracrine factors, which activate T cells and recruit them to the inflammation site but cannot activate Treg cells generation [20,33]. Preconditioning MSCs with IFN-γ alone was performed by other groups. However, they found that MSCs preconditioned with this cytokine highly express MHC (major histocompatibility complex) type I and II molecules that increase their immunogenicity and worsen their therapeutic properties. Therefore, we did not consider preconditioning with IFN-γ alone in our study [46].

We determined significant atherosclerotic lesions in the aorta of ApoE KO mice after staining with lysochromic stain within 60 days of feeding with an atherogenic diet.

To determine whether TNF-α-MSCs could affect T cells, we first studied the percentage of T cells after co-culturing with splenocytes of ApoE KO mice isolated for 8 weeks of treatment. We first observed that the percentages of CD4⁺ T cells and CD8⁺T cells in the spleens of ApoE KO mice increased in the TNF-α-MSC-treated group compared to the control. Our literature search did not reveal any studies discussing similar effects from TNF-α-preconditioned MSCs in an animal model of AS. We assumed that the immune response after treatment with TNF-α-MSCs was ongoing. Only data on using MSCs (untreated) confirmed those effects [21,24]. However, Frodermann et al. did not observe noticeable changes in the level of CD4⁺ T cells in the circulation and spleen after using untreated MSCs in a mouse model of AS [21]. Other researchers did not observe any changes in the percentage of CD8⁺ T cells after administering MSCs to AS mice [21].

Tregs have an important role in inhibiting the inflammatory processes in AS by mediating its immunosuppressive function and producing inhibitory cytokines such as IL-10 and TGF-β [47]. A small number of FOXP3+ Tregs were identified in atherosclerotic plaques [48]. Blocking of *FOXP3* leads to the progression of AS in mice, suggesting that FOXP3+ Tregs perform an athero-protective function [48]. Tregs reportedly demonstrate an athero-protective role by suppressing the functions of dendritic cells and Th1/Th2 (T helper 2) cells in mice with hyperlipidemia and immune deficiency [13,24,36]. In addition, IL-10 and TGF-β1, which are secreted by Tregs, limit the functionality of dendritic and Th1/Th2 cells in AS [13,24,25]. In our results, Tregs increased in the spleens of the TNF-α-MSCs group compared to the control. Our literature search did not reveal any studies discussing similar effects from TNF-α-preconditioned MSCs in an animal model of AS. Previous studies reported that using untreated MSCs can alter T cells through polarization from proinflammatory phenotypes (Th17 (T helper 17), Th1, and Th2), with a reduced production of IFN-γ, towards the anti-inflammatory phenotype (Tregs) with a high production of IL-10 [49]. According to Rohm et al., the lipid core of unstable plaques in human atherosclerotic lesions mostly contained Tregs, which were not present in the erupted zones of these plaques [48]. Furthermore, Wang et al. reported an increase in Tregs in ApoE^−/−^ mice after BM-MSC injection [25]. MSCs have different mechanisms of FOXP3⁺Tregs activation: through secreting IL-10, HLA-G5, IDO, PGE2, heme oxygenase-1, and TGF-β, and activating the T-cell receptor (TCR), which increases the ratio of Tregs [11,25,36,48]. Moreover, some studies have described the inhibitory role of Tregs in immune reactions along with their regulatory role in macrophage polarization in AS plaques [13,24,50]. Interestingly, MSCs significantly impacted AS inhibition by increasing the number and function of the CD4⁺CD25⁺FOXP3⁺ subset of Tregs, activating *FOXP3* gene expression, and reducing the number of macrophage foam cells [25]. The size of the atherosclerotic lesion reportedly increased when there was a deficiency of CD25⁺Tregs [25]. Furthermore, the quantity of FOXP3⁺Tregs in stable lesions substantially reduced, whereas proinflammatory T-cell and dendritic-cell levels were increased during AS development [51]. Li et al. reported that MSCs injection into experimental animals with AS promoted chemokine and cytokine production [52]. It also activated Tregs generation [20,52]. Lin et al. concluded that the role of either Tregs or macrophages is important in the regression of AS while using untreated BM-MSCs [22]. Thus, preconditioning MSCs is a promising approach to inhibiting the development of AS.

The effects of TNF-α-preconditioned MSCs on the Th1 subtype of T cells were not yet considered. Therefore, our results showed that TNF-α-MSCs slightly decreased the quantity of Th1 cells. The level of Th1 cells also decreased in the MSCs group, which is consistent with previously published studies [21,22].

Our study demonstrated that administering TNF-α-MSCs decreased the level of proinflammatory cytokines TNF-α and IFN-γ and raised the production levels of the anti-inflammatory cytokine IL-10 in the spleens of ApoE KO mice. Thus, administering TNF-α-MSCs decreased the development of inflammation in ApoE KO mice after 8 weeks of treatment. Previously published studies reported the effect of untreated BM-MSCs on AS development in mouse and rabbit models [11,13,24,25]. TNF-α, IL-6, and IL-10 production levels reportedly differed among themselves depending on the transplantation time of rabbit-derived BM-MSCs (untreated with cytokines) in a rabbit model of AS [25]. In addition, it was observed that MSCs lowered the production of IFN-γ by Th1 cells [25]. Moreover, IL-10 was accumulated and released moderately during immune response development [24]. Frodermann et al. also found an increase in IL-6 levels while using untreated BM-MSCs [21]. Furthermore, S-MSCs (untreated) decreased TNF-α production and increased IL-10 production, which depends on NF-kB pathway activation [52]. However, MSCs’ effect on the development of AS is still being studied. IL-6 can act as both a proinflammatory and anti-inflammatory cytokine depending on the target cell [53].

Our study showed that administering TNF-α-MSCs and MSCs (untreated) significantly decreased the spleen weights of ApoE KO mice. We believe this effect is mediated by a decrease in the production of proinflammatory cytokines TNF-α and IFN-γ.

The effects of TNF-α-MSCs on lipid levels in AS have not been well studied. However, MSCs (untreated) not only suppressed inflammation but also significantly reduced plasma cholesterol levels by lowering very low-density lipoprotein (VLDL) levels [21]. After administering TNF-α-MSCs to ApoE KO mice in the eighth week, total cholesterol and LDL levels decreased, whereas HDL levels slightly increased in the blood serum. Our results also showed that MSCs (untreated with cytokines) decreased total cholesterol and LDL levels, whereas HDL levels increased. We observed reduced plasma cholesterol in the fourth week of treatment using MSCs (untreated)]. It was previously reported that IL-10 over-expression reduced plasma cholesterol levels by lowering cholesterol and VLDL levels in mice with AS [24].

On the one hand, there was a significant decrease in lipoprotein lipase levels in the livers of mice injected with MSCs (untreated), which reduced the availability of free fatty acids for VLDL synthesis. Reduced VLDL metabolism was also found due to decreased Kupffer cell activation in mice after MSC (untreated) application [40]. Kupffer cells express mediators that promote VLDL secretion by hepatocytes. Moreover, lipoprotein lipase deficiency in macrophages reduces their VLDL uptake and oxidizes low-density proteins, thereby mitigating the course of atherosclerosis. In general, MSCs (untreated) lower VLDL levels and reduces inflammation [54]. It is known that IL-6 is one of the paracrine factors in MSCs. IL-6 plays an athero-protective role in the lipid processing system due to ABCA1 (ATP-binding cassette A1 protein) enhancement, which is involved in the outflow of lipids from macrophages, as well as a positive role in endothelial activation and inhibiting inflammatory cytokines [54]. One clinical study reported reduced levels of LDL cholesterol and triglycerides in patients with AS after Ad-MSC (adipose-derived, untreated) application [55]. In addition, exosomal miR-133 of BM-MSCs (untreated) suppressed expression of *ABCA1* and *ABCG1* (ATP-binding cassette sub-family G member 1) genes associated with cholesterol efflux from macrophage surfaces [56]. Gorabi et al. concluded that MSCs can reduce the aggregation of foam cells by decreasing cholesterol adsorption and enhancing high IL-10 production, which lowers the risk of developing AS [57]. By contrast, no changes in plasma lipid levels were reported after administering human amnion MSCs [41], S-MSCs, and human gingiva MSCs (hGMSCs) (all untreated) [50,52]. Thereby, the effect of MSCs likely depends on the source of the cells. Thus, MSCs can indirectly affect cholesterol metabolism through immune modulation by paracrine factors (IL-10 and IL-6) and exosomal microRNAs.

Prior to this study, the effect of TNF-α-MSCs on atherosclerotic plaques was not investigated. Our results based on the staining with Oil-Red-O of aortic cryosections with plaques demonstrated that TNF-α-MSCs reduced the development of AS in atherosclerotic plaques. Significant differences, however, were not found when compared with untreated MSCs. In our data, decreases in atherosclerotic lesion sizes in the untreated MSC group were similar to previously published studies when BM-MSCs and human amnion MSCs were used [21,41]. Moreover, macrophage content was reduced in mice modeled with AS after treatment with MSCs (untreated) because these cells can affect the polarization of macrophages from the M1 toward the M2 phenotype [24,32,41,52,58]. Administering MSCs (untreated) resulted in the size reduction in the plaques and depletion of lipid content in AS-modeled mice [24,58]. Thus, knowing that atherosclerotic plaques contain macrophages, all data show that MSC administration (untreated) to AS-modeled animals resulted in a regression of atherosclerotic lesions. Interestingly, unstable lesions (fibrous cap < 100 µm) contained predominantly dendritic cells, T helper cells, cytotoxic T cells, and NK (natural killer) cells. They also contained fewer Tregs than stable lesions (fibrous cap > 100 µm). Unstable lesions had a higher percentage of macrophages and T cells than stable lesions [7]. Transplanting MSCs (untreated) can balance unstable plaques in AS by immune regulation, altering matrix metalloproteinases that lead to collagen deterioration, and reducing the number of apoptotic cells [23,24,25]. Furthermore, Li et al. reported that S-MSC treatment diminished the size of the mice’s plaque in the aortic arch and limited the progression of AS [52].

## 5. Conclusions

In summary, our data demonstrated that TNF-α-MSCs contributed to the increase in Tregs and Th1 cells, decrease in the levels of proinflammatory cytokines TNF-α and IFN-γ, increase in anti-inflammatory IL-10 production, reduction in the spleens’ weights, decrease in total cholesterol and LDL levels, and increase in HDL levels. Thus, MSCs play an essential role in suppressing inflammation and sustaining atherosclerotic plaques.

Further research is necessary to determine the conditions for long-term favorable MSC effects and decipher the mechanism following MSC administration in different phases of AS.

## 6. Patents

A patent of the Republic of Kazakhstan (№. 2020/0278.1) was received on 30 April 2020 for the title “Method of preconditioning mesenchymal stem cells to enhance their anti-inflammatory properties”.

## Data Availability

The data presented in this study are available on request from the corresponding author.

## Supplementary Materials

The following supporting information can be downloaded at: https://www.mdpi.com/article/10.3390/cells12182262/s1, Figure S1: Phenotypical characteristics of MSCs. Figure S2: Feeding with an atherogenic diet induces the development of AS in ApoE KO mice. Figure S3: TNF-α-MSCs treatment reduces the development of AS. Figure S4: TNF-α-MSCs treatment reduces the development of AS.
