# Supplementary material for: TNF-α Preconditioning Improves the Therapeutic Efficacy of Mesenchymal Stem Cells in an Experimental Model of Atherosclerosis
Source: Cells. 2023 Sep 13;12(18):2262. doi: 10.3390/cells12182262 (PMC10526914; doi:10.3390/cells12182262)
Supplement: Supplementary file 1 [file cells-12-02262-s001.zip › cells-2458515-supplementary.pdf]

Supplementary Materials

TNF-α Preconditioning Improves the Therapeutic Efficacy of Mesenchymal Stem Cells in an Experimental Model of Atherosclerosis

Aliya Sekenova [1], Yelena Li [1], Assel Issabekova [1], Arman Saparov [2] and Vyacheslav Ogay [1,*]

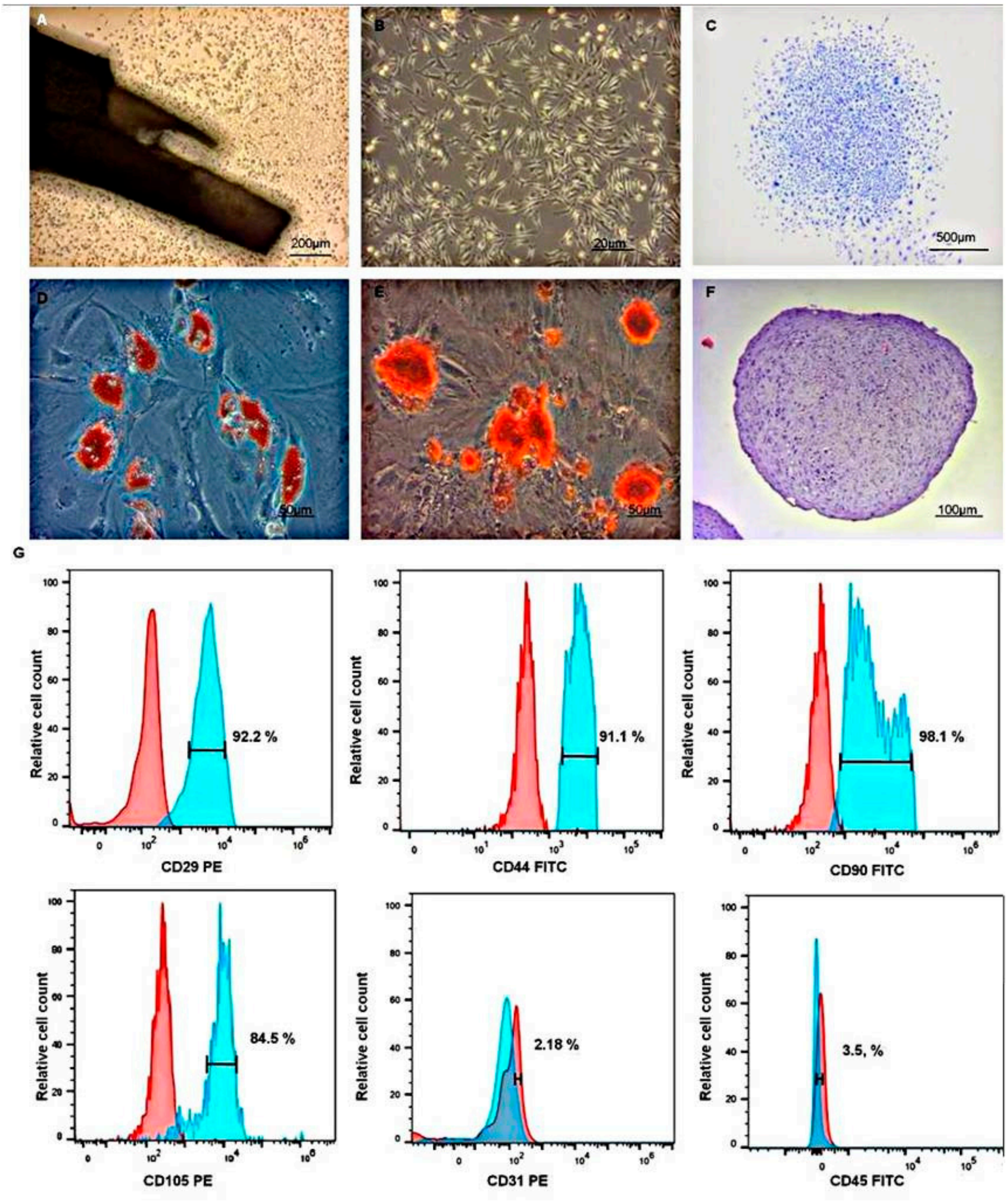

Supplementary Figure S1. Phenotypical characteristics of MSCs. MSCs were obtained from the compact bone of male C57BL/6 mice. **(A)** Representative image of fibroblast-like cells that migrated from the compact bone. **(B)** Representative image of MSCs (passage 2) with typical spindle-shaped morphology. **(C)** Representative image of the fibroblast-like colony of MSCs stained with crystal violet. **(D)** Representative image of the adipogenic differentiation of MSCs. Cells contain lipid droplets stained with Oil-red-O (orange). **(E)** Representative image of the osteogenic differentiation of MSCs. Cells contains the depositions of calcium stained with alizarin red S (orange). **(F)** Representative image of the chondrogenic differentiation of MSCs. A cross-section of cells' pellet after chondrogenic differentiation of MSCs stained with toluidine blue stain is demonstrated. **(G)** The expression of surface markers was analyzed by flow cytometry. Representative histograms are shown. MSCs, mesenchymal stem cells; C57BL/6 mice, C57 black 6 inbred strain of laboratory mice.

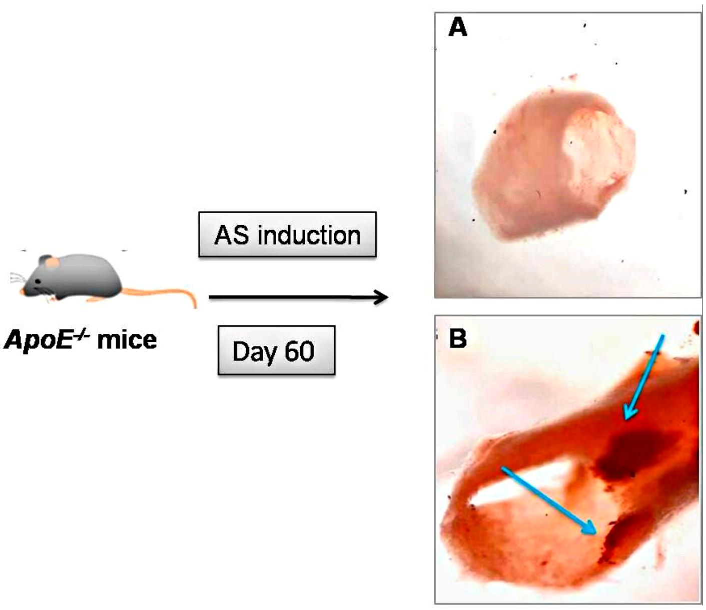

Supplementary Figure S2. Feeding with an atherogenic diet induces the development of AS in the aorta of ApoE KO mice. **(A)** Representative image of aorta section stained with Oil-Red-O (red) in the control group without atherosclerotic lesion. **(B)** Representative image of aorta section stained with Oil-Red-O (red) in the experimental group with the presence of an atherosclerotic lesion. Magnification, x200. AS, atherosclerosis; ApoE KO mice, mice with a knockout of *Apoe* (apolipoprotein E) gene.

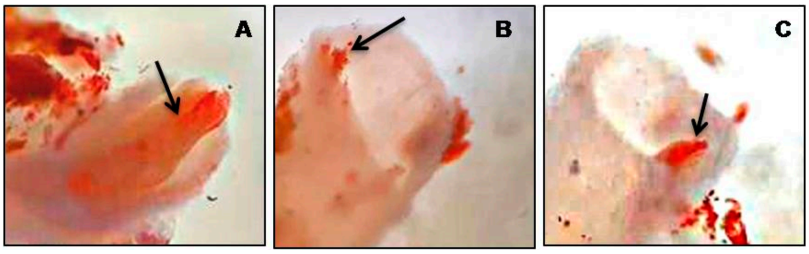

Supplementary Figure S3. TNF-α-MSCs treatment reduces the development of AS. **(A)** Representative image of the thoracic aorta with atherosclerotic lesion of the control (PBS) group of ApoE KO mice stained with Oil-Red-O (red). Magnification, x200. **(B)** Representative image of the thoracic aorta with the atherosclerotic lesion of the MSCs group of ApoE KO mice stained with Oil-Red-O (red). Magnification, x200. **(C)** Representative image of the thoracic aorta with the atherosclerotic lesion of the TNF-α-MSCs group of ApoE KO mice stained with Oil-Red-O (red). Magnification, x200. AS, atherosclerosis; ApoE KO mice, mice with a knockout of *Apoe* (apolipoprotein E) gene; TNF-α-MSCs, TNF-α preconditioned MSCs.

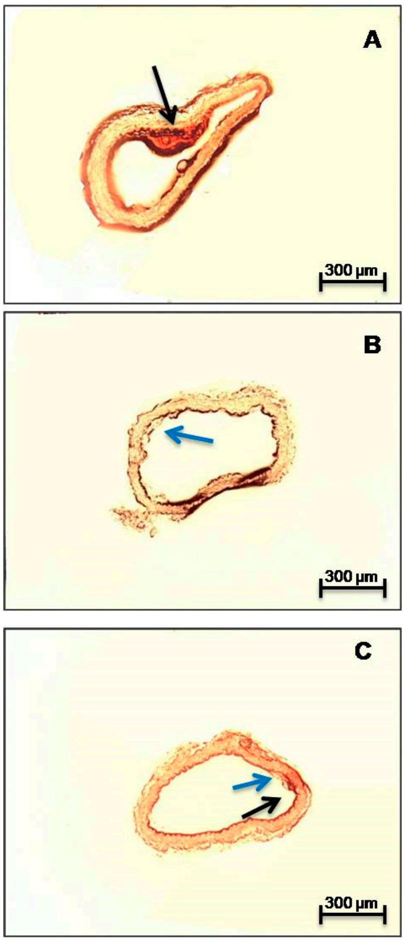

Supplementary Figure S4. TNF-α-MSCs treatment reduces the development of AS. **(A)** Representative image of the thoracic aorta with atherosclerotic lesion of the control (PBS) group of ApoE KO mice stained with Oil-Red-O (red). **(B)** Representative image of the thoracic aorta with the atherosclerotic lesion of the MSCs group of ApoE KO mice stained with Oil-Red-O (red). **(C)** Representative image of the thoracic aorta with the atherosclerotic lesion of the TNF-α-MSCs group of ApoE KO mice stained with Oil-Red-O (red). AS, atherosclerosis; ApoE KO mice, mice with a knockout of *Apoe* (apolipoprotein E) gene; TNF-α-MSCs, TNF-α preconditioned MSCs; Black arrow indicated the plaques with lipid content; Blue arrow indicated the plaques without lipid content.
